# Early Warning Systems for COVID-19 Infections Based on Low-Cost Indoor Air-Quality Sensors and LPWANs

**DOI:** 10.3390/s21186183

**Published:** 2021-09-15

**Authors:** Nikolaos Peladarinos, Vasileios Cheimaras, Dimitrios Piromalis, Konstantinos G. Arvanitis, Panagiotis Papageorgas, Nikolaos Monios, Ioannis Dogas, Milos Stojmenovic, Georgios Tsaramirsis

**Affiliations:** 1 Department of Electrical and Electronics Engineering, University of West Attica, 12244 Athens, Greece; msciot19007@uniwa.gr (N.P.); vcheimaras@uniwa.gr (V.C.); ppapag@uniwa.gr (P.P.); n.monios@uniwa.gr (N.M.); i.dogas@uniwa.gr (I.D.); 2 Department of Industrial Design and Production Engineering, University of West Attica, 12244 Athens, Greece; piromali@uniwa.gr; 3 Department of Natural Resources Management and Agricultural Engineering, Agricultural University of Athens, Iera Odos 75, 11855 Athens, Greece; 4 Computer Science Department, Singidunum University, 160622 Beograd, Serbia; mstojmenovic@singidunum.ac.rs; 5 Higher Colleges of Technology, Abu Dhabi Women’s College, Abu Dhabi 25026, United Arab Emirates; gtsaramirsis@hct.ac.ae

**Keywords:** air pollution, indoor air quality, early warning, COVID-19, air-quality sensors, internet of things, LoRa, wireless sensors networks, open-source

## Abstract

During the last two years, the COVID-19 pandemic continues to wreak havoc in many areas of the world, as the infection spreads through person-to-person contact. Transmission and prognosis, once infected, are potentially influenced by many factors, including indoor air pollution. Particulate Matter (PM) is a complex mixture of solid and/or liquid particles suspended in the air that can vary in size, shape, and composition and recent scientific work correlate this index with a considerable risk of COVID-19 infections. Early Warning Systems (EWS) and the Internet of Things (IoT) have given rise to the development of Low Power Wide Area Networks (LPWAN) based on sensors, which measure PM levels and monitor In-door Air pollution Quality (IAQ) in real-time. This article proposes an open-source platform architecture and presents the development of a Long Range (LoRa) based sensor network for IAQ and PM measurement. A few air quality sensors were tested, a network platform was implemented after simulating setup topologies, emphasizing feasible low-cost open platform architecture.

## 1. Introduction

According to the World Health Organization (WHO), clean air is a basic requirement of life. The quality of air inside homes, offices, schools, daycare centers, public buildings, health care facilities, or other private and public buildings where people spend a large part of their life is an essential determinant of healthy life and people’s well-being. Unfortunately, it is currently jeopardized due to various hazardous substances emitted from buildings, construction materials, and indoor equipment or due to general human activities indoors, such as combustion of fuels for cooking or heating, that lead to a broad range of health problems that may even be fatal [1,2]. Early in the 1990s, evidence of the effects of air pollutants on health was accumulating rapidly.

Short-term exposure to high levels of air pollution reduces life expectancy by roughly 8.6 months on average in the European Region by aggravating pre-existing respiratory and cardiovascular diseases [3,4]. Air pollution is referred to be the presence of contaminating particles dispersed in the air referred to as Particular Matter (PM) and gas within the atmosphere, such as carbon monoxide (CO), carbon dioxide (CO_2_), ozone (O_3_), nitrogen oxides (NOx), sulfur dioxides (SO_2_), ammonia (NH_3_), and Volatile Organic Compounds (VOCs), as well as some gaseous forms of metals. In Beijing, China, the presence and composition of organisms in the air was tested during a period of high smog. The sequences of several pathogens were identified that included viral particles (0.1% in both PM_10_ and PM_2.5_), and as the concentration of pollutants increased, the quantity of these pathogens increased as well [5]. In 2010, ambient air pollution, represented as annual PM_2.5_ deposition, accounted for 3.1 million deaths and roughly 3.1% of global disability-adjusted life years.

The Environmental Protection Agency (EPA) defines Particulate Matter (PM) as minute hydrocarbon particles, made up of joined aromatic rings, dispersed in the air for enough time to be diffused and transported. They are classified as PM_10_ or PM_2.5_ referring to their diameter of fewer than 10 micrometers or 2.5 micrometers, respectively [6]. PMs induce severe adverse cardiovascular and respiratory effects that are linked to their deposition in the lungs. Some PMs permeate through the air-blood barrier to extra-pulmonary sites, thus resulting in systemic inflammation [7,8], even affecting male fertility [9], as well as inducing neurodegenerative diseases [10,11,12]. Sancini et al. [13] revealed that prolonged exposure to high quantities of PMs induces major negative health effects, especially for the elderly.

Even at relatively low concentrations, the burden of air pollution on health is proven to be significant, involving daily mortality rate increases and admissions to hospitals [14]. Therefore in 2000, the World Health Organization’s Regional Office for Europe published a second edition of the WHO Air Quality Guides (WHO Regional Office for Europe, 2000), in close cooperation with the International Program on Chemical Safety, to effectively manage measures of air quality as a necessity to minimize health risks [15].

COVID-19, as well as other airborne viruses (CoV’s) named coronaviruses, are transmitted from person to person mainly through the respiratory tract and can cause mild to severe diseases, extending from the common cold to respiratory syndromes, such as MERS (Middle East Respiratory Syndrome) and SARS (Severe Acute Respiratory Syndrome) [11,16]. According to the thorough review of Comunian S. et al. [11], concerning the spread of viruses in the population referring to the effect of PM pollution, quite a few recent studies have analyzed whether the high and rapid increase in the COVID-19 contagion was correlated to a higher level of air pollution. Until now, China, Italy, and the USA experienced a high number of people infected by COVID-19, while all three of these countries suffer from a very high level of air pollutants thus leading recent studies to focus on these areas to find a possible correlation between air pollution and the COVID-19 contagion. Such findings have led to discussions related to the applications of breath analysis in the diagnosis of viral infections [17].

To evaluate the long-term exposition hypothesis, Pansini and Fornacca [18] investigated the geographical expansion of the infection and correlated it with the annual indexes of air quality, while analyzing different pollutants, such as ozone, carbon monoxide, PM_10_, PM_2.5_, sulfur dioxide, and nitrogen dioxide. The authors found eminent positive correlations in each country between air quality variables and COVID-19 infections and concluded that higher mortality was also correlated with poor air quality, especially, with high PM_2.5_, carbon monoxide, and nitrogen dioxide values. Recently, Wu et al. [19], estimated a profound positive relation for the United States between long-term exposure to PM_2.5_ and COVID-19 mortality rates.

These findings confirm the known relationship between PM_2.5_ exposure and many of the cardiovascular and respiratory comorbidities that significantly increase the risk of death in COVID-19 patients [19]. A position paper proposed by the Italian Society of Environmental Medicine (SIMA) considers PM as an important carrier contributing to the spread of COVID-19 [20]. The high, rapid spread of the COVID-19 virus in the Po Valley in Italy could be related to the high concentrations of PM present in Lombardy, as measured before the COVID-19 pandemic [21]. Deriving from Comunian, S. et al. [11], the Italian city of Bergamo proved to be the city with the highest number of people being infected, while concentrations of PM_10_ and PM_2.5_ vastly exceeded the permitted annual average values during January and February of the year 2020 there. In these same provinces, the COVID-19 contagion increased heavily and rapidly [11]. Many different studies have since provided corresponding evidence which confirms the above estimations. Frontera et al. [22], analyzed the air quality in Italy and China during the period of maximum COVID-19 virulence and found quite high levels of PM_2.5_ and nitrogen dioxide, just as Marteletti and Marteletti [23] concluded as well. Recently, by studying sequential image analysis, these authors hypothesized and proved that air rich in pollutants combined with specific climatic conditions may induce a longer presence of the viral particles in the air thus carrying airborne viruses farther, indulging an “indirect” diffusion. Setti et al. [24] showed that high-frequency concentration peaks of PM_10_ (over 50 µg/m^3^) accelerate the spreading of COVID-19, suggesting a “boost effect” for viral infectivity. Focusing on Milan and Rome they also found profound differences both in PM_10_ exceedances and COVID-19 spreading between Northern and Southern Italian regions. Zhu et al. [25] analyzed the relationship between the concentrations of six daily measured air pollutants, such as PM_10_, PM_2.5_, ozone, sulfur, carbon monoxide, carbon dioxide, and nitrogen dioxide and confirmed COVID-19 cases in 120 cities in China, concluding that there is a statistically significant relationship between short-term exposure to higher air pollution and an increased risk of COVID-19 infection.

Making use of air quality data from 25 cities in the European Union leads to an estimation that life expectancy may be increased by up to approximately 22 months in the most polluted cities if the long-term PM_2.5_ concentration is reduced to lower levels than those implied by the WHO annual guideline level [26]. Accordingly, the European Committee has implemented actions to monitor air quality levels to reduce air pollution and thus, health hazards in coordination with the World Health Organization. Internet links related to the available data and information on Europe’s air pollution are provided from the European Environment Agency (Air Quality Index) [27] which gathers data regarding air pollution from a wide range of sources. Information based on five different air pollutants is combined to show the current status of Europe’s air quality resulting in the European Air Quality Index that enables users to understand more about air quality within their premises while displaying up-to-the-minute data throughout the whole of Europe. Accordingly, such dedicated monitoring systems could be utilized indoors for enhancing Indoor Air Quality (IAQ). Utilizing several real-time Indoor Air Quality monitors, using feasible, low-cost air quality wireless monitors and sensors could significantly engage in active environmental monitoring and help reduce a vast variety of indoor air pollutants thus leading to improved breathable air within premises.

Taking such direct measures for alarm indications, sufficient infiltration and ventilation handled by such IAQ air quality monitors, a significant burden worldwide could be relieved from direct healthcare costs, premature mortality, significant disability, lost productivity, and various social consequences. Maniscalco et al. [28] carried out a study across two hospitals located in different Italian regions, to compare the accuracy of a COVID-19 rapid test with the accuracy of an exhaled condensate device. This device was based on a reverse transcriptase-polymerase chain reaction and the authors suggested using it inside airports or colleges for the rapid screening of a large population.

Therefore, it is highly recommended to introduce local monitoring systems inside buildings, including hospitals and schools, since there may be substantial variations in pollutants over a relatively small area. Thus, there are ongoing efforts all over the world by scientists and governments, to conduct large-scale IAQ monitoring. A variety of both commercial and research monitoring systems have been developed that have helped researchers monitor the IAQ. To this end, researchers have been focused on the IoT and smart sensor systems, which can gather and analyze data, and perform both monitoring and warning functions. Finally, there is an improvement of IAQ and therefore, of the health and comfort of people who live or work inside a building. The price and the capabilities of IAQ monitoring systems vary depending on their structure and the coverage area.

Ha et al. [29] propose an air quality management system, merging the indoor air quality index and humidity measurements into an enhanced IAQ index, by using real-time sensor data. The air pollutant levels can be measured by a network of wasp sensors, while IAQ and humidity data are fused, with the help of an extended fractional-order Kalman filter. According to the obtained enhanced IAQ index, overall air quality alerts are given in a timely fashion to provide an accurate prediction. To evaluate this method fractional-order modelling was used as well as a control toolbox. Also, a case study has been analyzed to prove the effectiveness and validity of this approach.

A portable low-cost IoT IAQ monitoring system, with 30 h of battery life, has been proposed by Esfahani et al. [30]. This monitoring system was designed for the monitoring of total Volatile Organic Compounds (VOCs), Carbon dioxide (CO_2_), Particular Matter (PM) 2.5, Particular Matter (PM) 10, temperature, humidity and luminance of an area. It can be used to monitor air quality in large-scale networks for Smart Cities. Moreover, the portable unit can be used either for real-time measurements or hourly and daily averaging. It uses low power modes, and it integrates with an easy user engagement, custom Blynk smartphone app. The device calculates a qualitative air quality index and therefore, environmental data are used by the system to introduce recommendations, such as increasing and decreasing ventilation or managing activity levels, to improve air quality.

Asthana et al. [31] devised a bolt IoT system that provides pollution level information directly to a smart device in real-time. In addition, the authors have also suggested some precautionary measures to improve students’ air quality, which is connected with their health and can positively impact their academic performance. Their system has two main Air Quality sensor units and a Bolt-based IoT board to monitor the air quality in real-time. The processed data is stored on a cloud computing platform. The system generates an alert when the air quality deteriorates beyond the permissible limits, and preventive measures may be implemented to reduce potential risks for students and staff. Moreover, they analyze the causes of increased pollutants, to benefit people endangered by significantly high concentrations of air pollutants.

Kaliszewski et al. [32], during the COVID-19 pandemic, compared low-cost PM sensors, for indoor air monitoring. Their study showed that both the intensity of a room’s usage and the number of persons inside the room had an impact on PM levels.

From the aforementioned literature review, we conclude that air pollution has become a major concern and its effects may lead to major health problems. IAQ has been largely ignored up to now, but as we spend plenty of time indoors especially in the pandemic era of COVID-19, it is crucial to monitor the air we breathe daily. We should take appropriate measures promptly, to measure and analyze the parameters of the air. Thus, the development of real-time air quality monitoring is essential. Ladekar et al. [33] propose an air quality monitoring module to deal with this problem. They introduced an IoT project, where IAQ is measured by using various sensors. IAQ data are pushed to an MQTT broker and are stored on Cloud databases. Furthermore, to achieve better visualization, graphs are plotted in real-time through Amazon Web Services (AWS) and users are alerted if the indoor air quality is poor.

Another approach that is gaining popularity is the Web of Things, where web architecture and web standards are used as a framework to create IoT applications. Esquiagola et al. [34] presented an IoT platform for monitoring indoor air quality, which is based on the Web of Things concept by using the CoAP protocol to collect IAQ data from sensors. They have also developed a hardware platform that uses custom hardware, firmware, and software to present a complete solution, including visualization of results.

To maintain IAQ, Nasution et al. [35] proposed a way to regularly monitor several parameters that can affect air quality. The development of sensor and monitoring technology based on IoT (Internet of Things) helped them to design an automatic monitoring system that receives data from devices periodically. This supports the design of a system that can periodically monitor indoor air quality conditions. In their study, they have designed a portable air quality monitoring system using the ESP32 as a controller and several sensors to measure air quality. The final result is a system that can monitor temperature, humidity, dust particles, and other polluting gases, such as H_2_S, NH_3_, CO, NO_2_, and SO_2_.

Zhi et al. [36] designed a monitoring platform with sufficient mobility for the places where monitoring stations are not easy to install. This mobile and portable monitoring platform with promoted sensors was based on unmanned aerial vehicles (UAV. Furthermore, this UAV platform can generate useful information for controlling indoor air quality inside buildings. To meet the indoor inhabitants’ health and comfort standards, a fuzzy control method was proposed to control the actuators of the air cleaner and ventilator. They aimed to build and improve an intelligent environment that meets the comfort level with an acceptable IAQ.

Motivated by the COVID-19 pandemic, we have introduced a low-cost, flexible, reliable, and scalable IoT-based system, to monitor and warn people, about air-borne infections. These infections are influenced by many factors that increase indoor air pollution. We focus on the parameters that determine the IAQ of both small and large buildings like schools, hospitals, homes, offices, etc. The most relevant parameters are the PM levels. Increased PM levels may cause a lot of damage to health as they may adsorb other toxic substances. Our proposal includes the implementation of an open-source platform based on the development of a Long Range (LoRa) sensor network, which measures the PM levels and monitors the IAQ in real-time.

## 2. Materials and Methods

**Figure 1:**
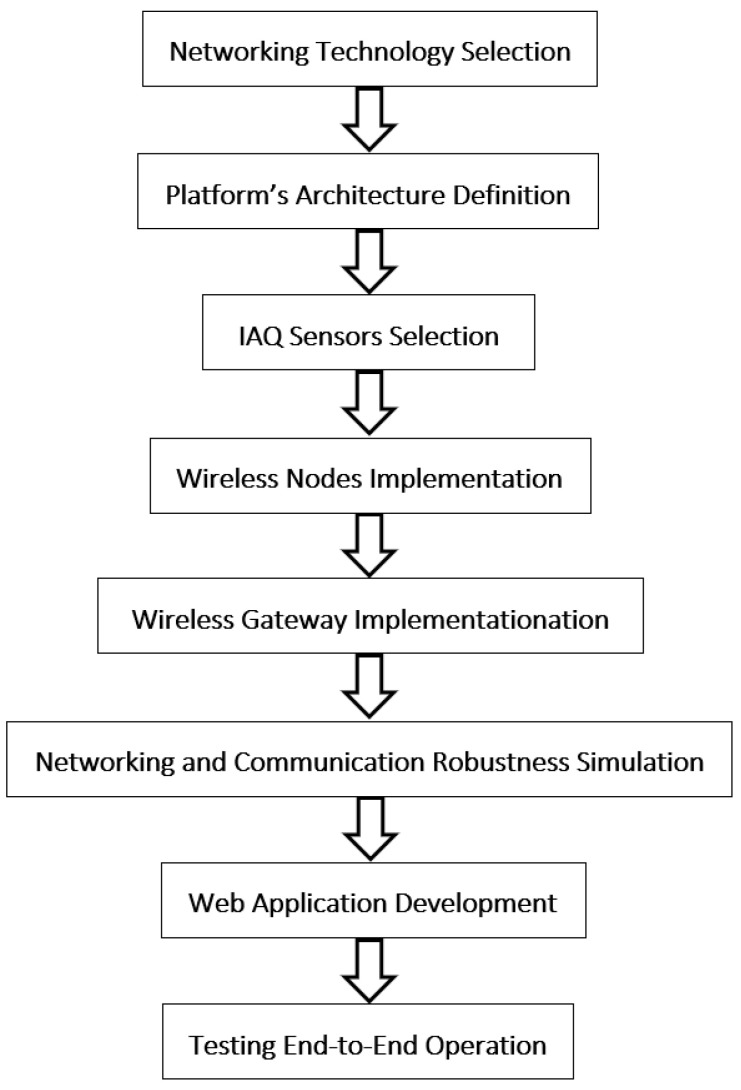
Methodology followed for realizing the proposed early warning system for COVID-19 infections based on low-cost Indoor Air Quality (IAQ) sensors and LPWANs.

Since the need for IAQ control to support early warning systems for airborne viruses was sufficiently justified in the introduction section, this section describes the methodology and materials used for the development and implementation of an IAQ wireless sensors network platform emphasizing the low-cost, low complexity, open-source and scalable properties. Specifically, Figure 1 presents the methodology we followed to meet our goal.

Additionally, we decided to implement just one piece of IAQ wireless sensor node, instead of many, to keep the cost of experiments low and because the networking and communication robustness simulation efficiently guarantees the demonstration of large deployment configuration behaviors. Several sensor nodes will be produced in the near future to investigate the air-quality control for specific use-cases in particular indoor environments in large public and private buildings. In any case, the use of a single wireless sensor node can support the testing of the end-to-end operation of the proposed platform as is reported in the following sections.

### 2.1. Indoor Low Power Wireless Network Communication Technologies

In the last years, cellular wireless technology (LTE) has been used for connecting sensor devices to the web. However, this technology has some limitations, because it is too costly to purchase and deploy. Also, it is often overly complex for simple sensors that do not send data very often and do not require significant bandwidth. Finally, cellular technology has expensive and complicated provisioning and management plans, that result in higher costs for the massive deployment of IAQ wireless sensors. There are alternatives to cellular technology, such as WiFi, Zigbee, and Bluetooth Low Energy (BLE). They can transfer large amounts of data over the internet, but they are limited by short data ranges and heavy power consumption. Moreover, they face some built-in complexities. For those reasons, we have chosen a new class of wireless technologies that have been built specifically for short-range low-bandwidth digital data transfer. Low Power Wide Area Networks (LPWAN), such as LoRa, Sigfox, and Narrow Band Internet of Things (NB-IoT) are the leading emerging standards in wireless sensor deployment.

Due to our goal being the use of open-source solutions providing on-premises minimum complexity for provisioning and massive deployment without vendor lock-up, we opt for LoRaWAN [37,38,39], which uses low-cost gateways (normally open-source) pushing the complexity to the higher levels of the network architecture while it is an on-premises suitable solution with open-source solutions from the sensor node to the gateway and the network operation servers.

On the other hand, base stations, maintenance, and operation/upgrading costs for NB-IoT are orders of magnitude higher than LoRaWAN, therefore, on-premises deployments are very expensive leading to complex and expensive implementations for massive deployment plans in coordination with mobile operators.

Similar to the NB-IoT technology, Sigfox is a patented technology without the possibility of on-premises deployments leading to dependencies that are not open-source.

LoRa that is the physical layer of the LPWAN technology is one of the most promising wireless technologies for IAQ sensor networks. It is a patented wireless communication protocol that achieves low-power consumption long-range transmissions, reaching over 10 km in line-of-sight situations, while trading off data rate with time-on-air. LoRa uses unlicensed sub-GHz radio bands, including 433, 868, and 915 MHz, thus having its duty cycle regulated by regional authorities. Due to its low transmission bandwidth, LoRa suits applications where transmissions are sparse and payloads are relatively small. Metering applications of noncritical parameters like weather meters, air quality, or other environmental meters, and also in animal tracking, smart agriculture, and connected farms are well suited to this approach [40,41].

LoRaWAN is characterized as a Low Power, Wide Area (LPWA) networking protocol designed to wirelessly connect battery-operated ‘things’ to the net in regional, national, or global networks, and targets key Internet of Things (IoT) requirements, such as bi-directional communication, end-to-end security, mobility, and localization services [42].

**Figure 2:**
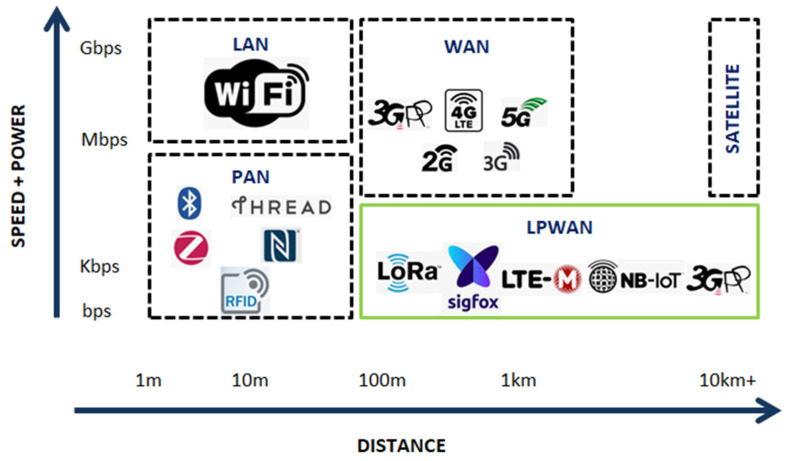
LPWAN compared with the popular LAB, PAN and WLAN existing technologies in terms of networks’ distance, data speed and power consumption properties.

Therefore, LoRaWAN is a member of the LPWAN category, as seen in Figure 2, which is suitable for connecting objects implementing significantly autonomous applications. LoRa makes use of ISM free-use frequency bands, shared with other wireless technologies. So, it is obliged to comply with defined usage rules, particularly concerning the transmission power, the duty cycle, and therefore, bandwidth. The most common use cases of LPWAN networks are Smart Cities, connected industries, and isolated data measurement [43].

### 2.2. Reference Architecture and Deployment Strategy

In the present work, we make use of the LoRaWAN network architecture being deployed in a classic star-of-stars topology in which gateways relay messages between the end-devices, i.e., the Indoor Air Quality (IAQ) sensor nodes and a central network server.

**Figure 3:**
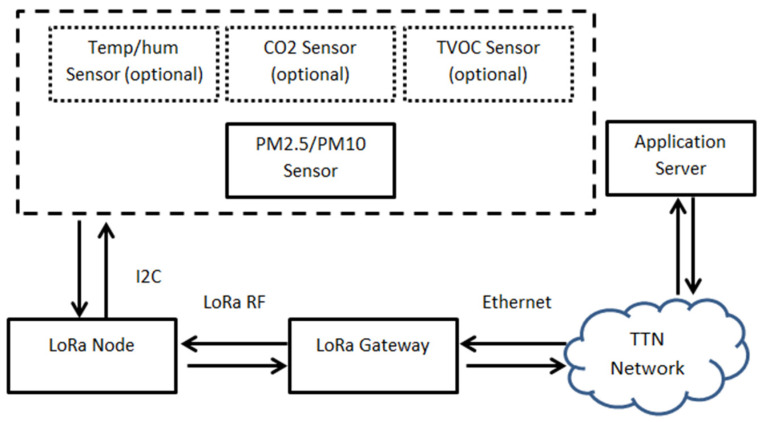
Block diagram of the proposed system’s architecture.

The gateways are connected to the network server, the TTN in our case, via standard IP connections and act as a transparent bridge, simply converting RF packets to IP packets and vice versa. The wireless communication takes advantage of the Long-Range characteristics of the LoRa physical layer, allowing a single-hop link between the end-device and one or even many gateways according to LoRa Alliance specifications [42]. The full block diagram of the platform is depicted in Figure 3 below.

### 2.3. IAQ Sensors

The choice of the appropriate sensor probably is the most significant factor for a reliable, accurate and sustainable IAQ scheme. Some sensors are suitable for indoor and others for outdoor use. The goal is to implement indoor low-cost, readily available sensors which provide accurate readings. For this reason, there has been a growing market for reliable, low-cost air quality sensors that have been developed to measure a wide range of pollutants.

Sensors that measure multiple pollutants may be reliable for some pollutants but not for others. Size and feasibility are to be kept in mind as well. For the above reasons, we decided to use the Sensirion SPS30 sensor for our experiments. Recently developed solid-state sensors give on-site readings in near real-time so there is no need to go to a lab. Table 1 quotes the sensors used in this work.

The characteristics of the sensors tested appear in Table 2, Table 3, Table 4 and Table 5. The tables have been filled-in with the maximum available information that exists on the world-wide web.

The following paragraphs present a list of highlighted observations and characteristics according to the technical data of the various studied devices, along with comparing sensor characteristics against our project goals.

1. TVOX-CO_2_ Gas sensors group
  - Sensirion SVM30

This device provides two complementary calibrated air quality output signals and features a dynamic baseline compensation algorithm, containing both Set and Get baselines and on-chip calibration parameters. A CO_2_ equivalent signal (CO_2_eq) is provided as an output by one single sensor chip. The sensing element is robust against contamination by siloxanes present in real-world applications which ensures long-term stability and low drift. It measures temperature and humidity as well. It is equipped with a widely used I^2^C connection. It poses a wide range of accurate measurements. Calibration memory and baseline values are stored within this device and recalled whenever needed through the bus, thus, enabling flexible designs, reliable and adequately accurate readings for indoor air quality sensor nodes.

  b. Bosch BME680

This device measures temperature, humidity, and atmospheric pressure besides providing directly an IAQ factor indication on a scale of 1 to 5 which is most convenient for use in IAQ sensor nodes. It utilizes ISP and UART connection ports while posing low energy consumption and requires a low voltage power supply thus suitable for small battery-powered setups. It stands well for enhanced stability during its service time.

  c. Renesas ZMOD4410

This sensor provides estimation values of Total Volatile Organic Compounds (TVOC’s) concerning the total amount of all VOC gases in the air, utilizing artificial intelligence (A.I.) algorithms for accurate and sustainable readings. It stores module configuration and calibration data to set itself under its environment algorithmically in 60 min which enhances accuracy and flexibility to environmental conditions. It may activate alarms that indicate the level of acceptability of odor emissions. Its I^2^C connection, small dimensions, low power consumption, and low voltage supply make it easy to use in small versatile battery-powered devices. It effectively withstands the effects of siloxane in very dusty and humid environments. That makes it a reliable device in the long term.

2. Particulate Matter sensors (suspended particles) group
  - Sensirion SPS30

This optical PM sensor measures a wide range of particulate matter (PM) scattered in the air like dust and other particles, ranging from PM_1_ to PM_10_ sizes and 0.3–1000 μg/m^3^. Its measurement principle is predicated on laser scattering.

It exports measured values of mass concentration in micrograms per cubic centimeters (μgr/m^3^) and the number of suspended particles per cubic centimeters (#/cm^3^) with adequate accuracy and relatively small drift per year. Air movement is required and is generated via an internal fan that unfortunately consumes energy. Even more, air movement leads to accumulated dust, therefore, cleaning of the fully operating internal fan is eminently required once a week.

It comes equipped with UART and I^2^C connections and thus, is approved for general commercial use. According to specifications, reliable operation is to be expected for 10 years of service life due to contamination resistance technology posing a drift of merely 1.25% per year. It has small dimensions and we have chosen it because it is the best solution for applications where size is of paramount importance, like wall-mounted or compact air quality devices [44]. This device could be an option for small flexible PM measurement nodes.

  b. Omron B5W-LD0101-1/2

This device emits electric pulses when it detects particles making use of light dispersed on these particles. According to the terminology specifications, the range of detectable particle sizes lies between 0.5–2.5 μm which corresponds to particles designated as PM_2.5_. Indicative smoke and dust measurements may be recorded. It requires an external power supply for calibration to a specific scale. It is prone to power supply ripple as it is an analog circuit. The device itself proves to be dependent on enhanced amounts of energy consumption although flexible and adaptive to a variety of environmental conditions depending on calibration conditions.

Referring to Table 2, Table 3 and Table 4, we may conclude that the vast majority of the sensors mentioned, provide reliable and accurate measurements for gases, PM, and even combine such measurements to calculate an IAQ factor. These outputs correspond to IAQ indexes outlined by the World Health Organization and the European Environment Agency [1,14,27]. Concerning the costs of the sensors, we addressed the site of the authorized global distributor of semiconductors and electronic components Mouser Electronics (Table 5) to justify the fact that these sensors are available in the open market reasonably priced.

Finally, lightweight, small and low-cost sensors make low-cost, standalone, lightweight applications, such as IAQ feasible. Furthermore, the fact that the existing IAQ sensors support one, or more, of the most popular and easy-to-implement serial data interconnection interfaces (Table 5), i.e., the I^2^C, SPI, and UART, offer integration flexibility to heterogeneous, low-cost platforms, thereby removing hardware dependencies leading to even more flexible and low-cost IAQ sensor applications.

### 2.4. Open-Source Software Applications’ Development Toolchain

According to support the use of open-source software tools, we have selected to use some of the most popular and effective solutions. Specifically, we adopted InfluxDB for web database development and Grafana for data visualization and user interfaces. On the other hand, as it regards the low-level programming that is the development of firmware for the IAQ wireless sensor nodes and gateways we adopted MicroPython because this is a hardware-agnostic programming approach. The Tings Network (TTN), a free-to-use and open-source tool has been selected for the LoRaWAN Network Server.

## 3. Results and Discussion

### 3.1. LoRaWAN-Based Indoor Air Quality Wireless Sensors Implementation

**Figure 4:**
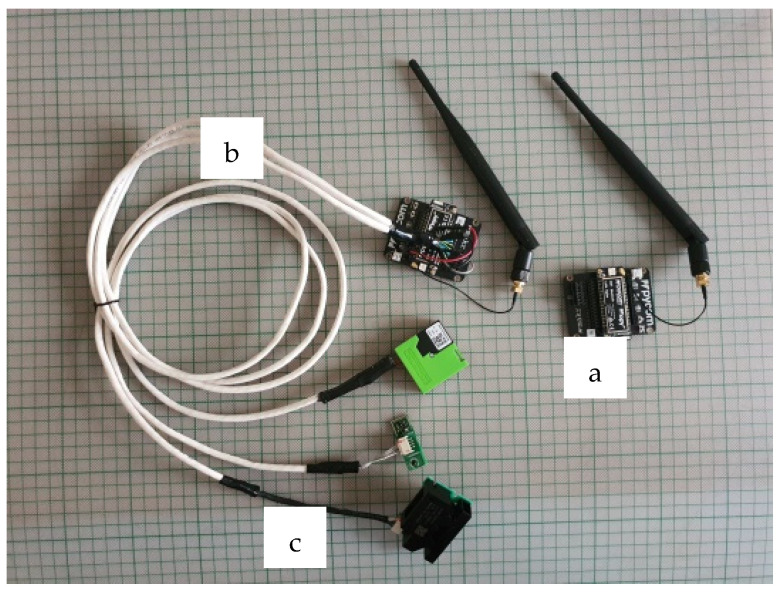
Scheme of the experimental setup of the LoRa sensors network based on a LoPy4 LPWAN gateway with a Pymark expansion board and the LoRa antenna mounted (**a**), LoPy4 node inserted in Pymark expansion board (**b**) and Air Quality sensors connected with LoRa node (**c**).

To monitor the IAQ, we have implemented a firm, low-power consumption, and scalable wireless sensor network (Figure 4) which is composed of low-cost LoRaWAN gateways (Figure 4a), LoRaWAN nodes (Figure 4b), and PM sensors (Figure 4c). We use a LoPy4 gateway and a LoPy4 node with a Sensirion SPS30 PM sensor to monitor and control the air quality.

The LoPy4 is a flexible network MicroPython-enabled development board that may be the perfect enterprise-grade IoT platform for our connected Things. With the most recent Espressif ESP32 chipset, utilizing a System-on-Chip (SoC) implementation [45], the LoPy4 offers us a combination of power, friendliness, and adaptability which allows us to create and connect our things everywhere.

The LoPy4 is used in the current project both as a low cost out off-the-shelf LoRa nano gateway, as well as a LoRa node importing data from connected sensors, while generally this may be used as a multi-bearer development platform suitable for all LoRa, Sigfox, WiFi, and BLE networks around the globe. These characteristics led us to choose this specific IoT platform for our experiment.

Both LoPy boards were programmed with MicroPython and other Pymakr plug-ins enabling fast IoT application development, easy in-field programming, and further resilience with network failover. We also have the option to configure the LoPy4 in raw LoRa mode to send packets directly between LoPy4′s [46].

**Figure 5:**
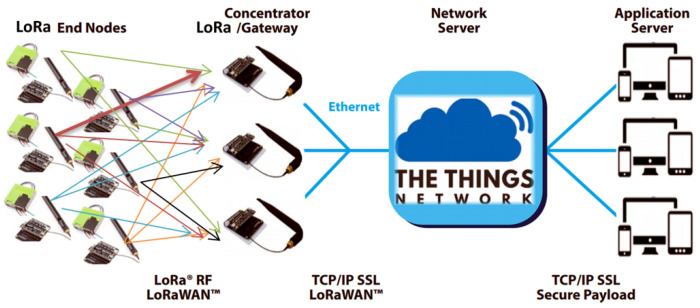
LPWAN LoRa utilized architecture and network topology.

Furthermore, our network uses The Things Network (TTN) Cloud server for data storage. We have also built a PHP script for storing the data into an Influx Database to visualize them on a Grafana server. A front-end web application has been developed to access and visualize the data stored in the Grafana server. Figure 5 depicts a scheme of the network topology, with the various hierarchical layers from the End nodes, the Gateways/Concentrators, the network server and the application server.

**Figure 6:**
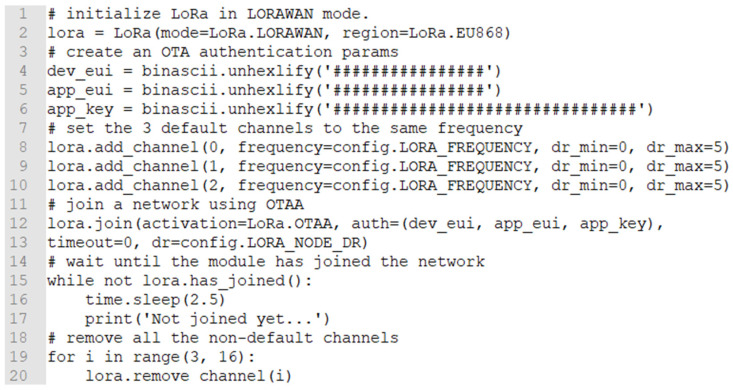

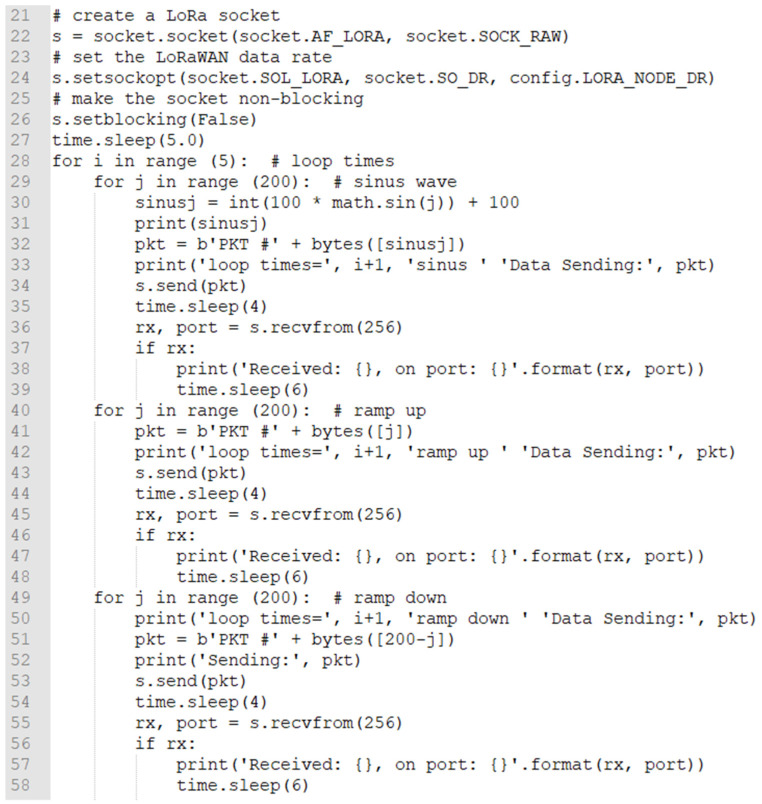
Part of the initialization and parameters configuration of LoRa-enable LoPy4 nodes.

In the snippet that follows, we provide an overview of the code implementation for the Lora-enabled LoPy4 node. The code is written in MicroPython using the PythonMkr development environment and it is ultimately stored as firmware in the LoPy4 node’s non-volatile program memory. In this specific part of the code, we can see the LoRa node’s initialization, the over-the-air authentication (OTAA) (Figure 6 lines 1–20), and the algorithm for the creation of a non-blocking socket (Figure 6 lines 21–58). It is apparent how easy is to configure all the critical operating parameters of the LoRa nodes.

In OTAA activation, a LoRa node performs a join procedure with a LoRaWAN network where a dynamic device address (DevAddr) is assigned to the end device, and root keys are utilized to derive session keys. The DevAddr and the session change when a new session is established. While the joined () routine is called, the server blocks until data are available for our socket. Problems will arise as soon as the server joins multiple sockets simultaneously or when the server wants to perform other tasks. These problems can be avoided, by configuring the socket as non-blocking.

### 3.2. Networking and Communication Robustness Simulation

It is essential to determine whether it would be feasible to use only one gateway instead of many gateways to efficiently address all sensor nodes in a building. The location of the gateway in a typical building should affect the performance of the network. To study the performance of such a network setup we went through simulation procedures.

To simulate a LoRa-based wireless sensor network of our IAQ nodes, we considered scenarios that require IAQ measurements implemented on the NS3 simulator making use of the LoRaWAN open-source module for NS3.

**Figure 7:**
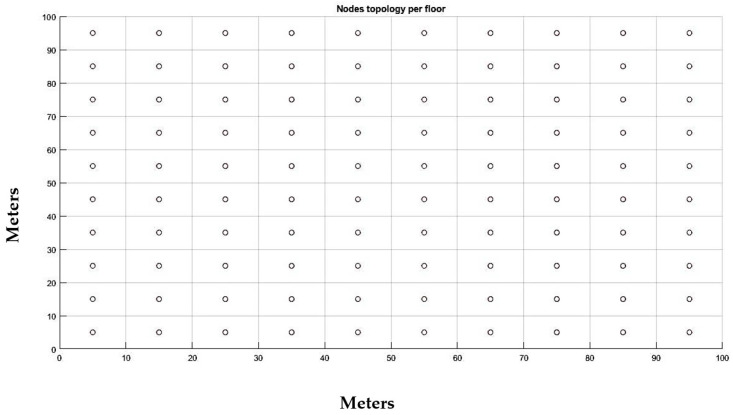
IAQ node deployment topology per floor.

**Figure 8:**
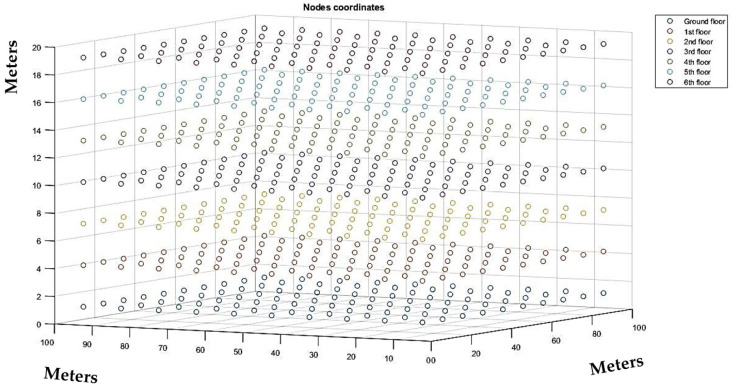
Topology of all installed IAQ nodes in the building in all three dimensions.

Next, we present the scenario that was implemented as well as the results of the Packet Delivery Ratio (PDR) that were taken from the simulation. We considered a seven-floor building made of concrete as the basis of our scenario. The dimensions of the building were 100 m × 100 m × 21 m, thus each floor was 3 m tall. We considered that there were 100 rooms per floor, with each room having dimensions 10 m × 10 m × 3 m. Finally, we considered that each room in the building faces inwards to at least one corridor through a door and all external walls have windowpanes as well as the masonry partitions, placed in high positions. The building could represent a hospital in which the IAQ nodes could be installed to measure the IAQ of each room. For this simulation, we did not consider any possible metal or plastic installation, machinery, or any other obstacles within the building. Then, we considered that an IAQ node will be installed in every room. The nodes were installed at the center of each room, 1.2 m above its floor. In total, there were 700 nodes installed in the building. In Figure 7, the topology of each IAQ node per floor is depicted, whereas in Figure 8 the topology of all 700 IAQ nodes that were installed in the building can be seen in three dimensions.

Each IAQ node was then programmed to sample a sensor every 5 min. A 20-byte-long message, which contains the number of the room and the value taken from the sensor with two-digit decimal precision, was then harvested from each sensor and was used as the payload of the node. For example, if a PM_2.5_ value of 33.67 μg/m^3^ in sequence with a PM_10_ value of 40.5 μg/m^3^ and a Total VOC value of 200 ppm, is received from a sensor node placed in room 39, the payload of the message shall be the format of “<Node>, <Val1>, <Val2>, <Val3>” (quotes characters starting and ending the data packet are not transmitted). So, in this case, the particular payload is “039,0033.67, 040.50,0200.5”.

To follow the EU’s LoRaWAN regulations, we used an online Airtime Calculator to decide the proper spreading factor of the node based on the payload, its header size, and the maximum number of allowed messages per day for that payload. In our case, given that we have a transmission frequency of 5 min, hence 288 transmissions per 24 h, we saw that we can only use a spreading factor of seven on our nodes because it allows up to 417 transmissions of 20-bytes-long plus 13-bytes-long header messages per day, whereas a spreading factor of eight allows up to 224 transmissions per day for the same payload and header, and each spreading factor after it is even less flexible. The bandwidth was set to be 125 kHz.

**Figure 9:**
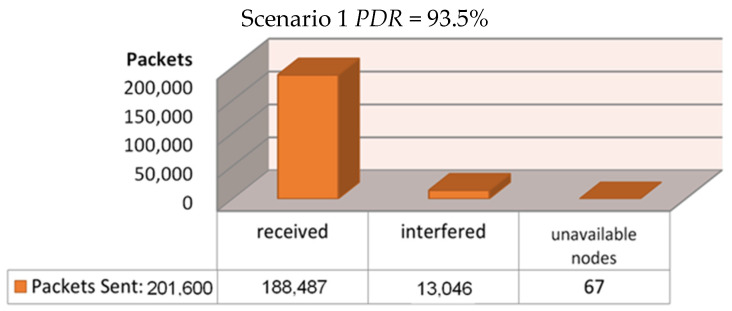
Scenario 1—Regarding the setup’s effectiveness once installing the gateway at the geometrical center of the building.

**Figure 10:**
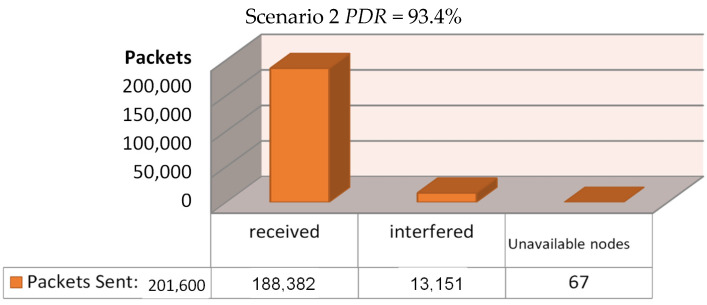
Scenario 2—Regarding the setup’s effectiveness once installing the gateway at the roof of the building.

**Figure 11:**
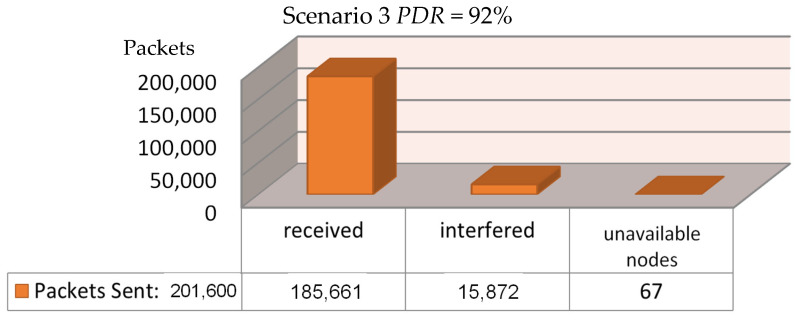
Scenario 3—Regarding the setup’s effectiveness once installing the gateway at the roof of a neighboring building.

The simulation script then ran three different scenarios, based on three different locations for the installed gateway, to decide where we are going to have a higher Packet Delivery Ration (PDR). The first scenario had the gateway installed inside the building, exactly at its center (Figure 9). The second scenario had the gateway installed at the roof of the building, at coordinates 50,50 as we see in Figure 10, whereas the final scenario had the gateway installed at the roof of a duplicate neighboring building—at its corresponding 50,50 coordinates—which was separated by a 7.5-m-wide road from the main building (Figure 11). Each simulation loop simulated the network behavior for 24 h. The simulation returns the total amount of packets sent in the network from each node to the gateway (or gateways, if more than one installed); the number of the successfully received packets; the number of the interfered packets; the number of packets lost because of lack of receiving nodes available to communicate with the gateway; the number of packets lost from the gateway due to low signals below receivers sensitivity; and the number of packets lost by a busy transmitting gateway. We can calculate the Packet Delivery Ratio by dividing the number of the successfully received packets by the total amount of packets sent:

$$PDR=\frac{Packets\;Received}{Packets\;Sent}$$

For the first scenario, where the gateway was installed at the geometrical center of the building, a PDR of 93.5% was achieved as can be seen in Figure 9. We may notice that only 6.5% of all transmissions were interfered with and lost, and merely 67 out of 201,600 messages were lost because of unavailable node connections with the gateway as pointed out by the simulation program, and we have zero losses due to low sensitivity or due to a busy transmitting gateway. For the second scenario, where the gateway was installed at the roof of the building, the PDR that was achieved reached the amount of 93.5%, as can be seen in Figure 10. Here, we noticed a 6.5% loss because of transmission interference, 67 out of 201,600 messages were lost because of unavailable node connections with the gateway due to a busy transmitting gateway, and again zero losses due to low sensitivity. Finally, for the third and final scenario, where the gateway was installed at the roof of a duplicate neighboring building, a PDR of 92.0% was achieved as can be seen in Figure 11. Only 8.54% of all transmissions were interfered with and lost, 67 packets lost because of unavailable node connections with the gateway, and zero losses due to low sensitivity or from a nearby transmitting gateway.

It is quite evident that both the first and the second scenarios, where the gateway is installed in the center or the roof of the building, provide adequately firm data transfer with minimal losses of any kind. Thus, it is mostly eminent that when the gateway is placed in the construction center of the building or even at the top floor of the building every single IAQ node is adequately covered, meaning that all IAQ nodes are available to be addressed and their aggregated data can be relayed to the central network server. Otherwise, more gateways should be placed after undergoing similar simulation processes to determine the best position to install them, leading to high costs and installation efforts.

### 3.3. Implementation of the LoRaWAN Network Server

The Things Network (TTN) Stack is known as a complete LoRaWAN open-source server stack designed for a spread of deployment scenarios that additionally support all existing LoRaWAN versions and operating modes (A, B, and C, and one regional parameter). The microservices architecture enables the separation of various concerns, distributions, scaling, and advanced interoperability with other systems. It has Join Servers that store LoRaWAN keys and safely issue session keys to the Network Server and Application Server. This decouples secure storage from packet routing, allowing us to host Join Servers on-premises while using Hardware Security Modules (HSMs) to remain in full control of the security keys. The Application Programming Interface API-first design offers open-source Remote Procedure Calls gRPC, Hypertext Transfer Protocol (HTTP), and Message Queuing Telemetry Transport (MQTT) data planes, and gRPC and HTTP control plane integrations. It enables the management of users, organizations, gateways, applications, and devices, and interacts in real-time to streaming uplink, downlink, and low-level processing events. The microservices architecture allows for replicating instances of specialized services and distributing services in clusters to make highly scalable and highly available global LoRaWAN networks. The monitoring and diagnostics features are also very useful when developing new LoRaWAN devices and applications, as we can run the stack as a lightweight single binary on our development machine and see exactly how our device behaves. In our implementation, all uplink messages that include sensor data arrive at the TTN application server through a payload function. After that, there is a data decoding function that decodes sensor measurements and lastly, the sensor data are set in JSON format to match the requirements of the front-end application. The sensor data are stored through the Data Storage integration where they can be downloaded via HTTP. Furthermore, there is an integration that stores data into an Influx database and another one that permits visualization within the Grafana server.

### 3.4. Sensors’ Database

**Figure 12:**
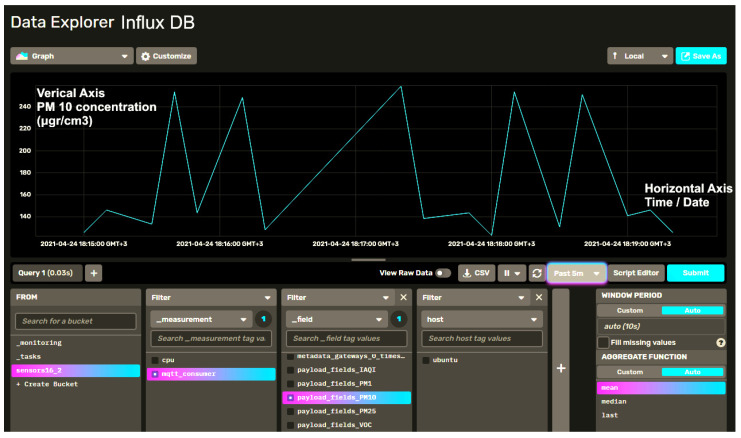
InfluxDB data Explorer visualizing PM_10_ accumulated data in time series for the depicted time interval of 5 min.

We have integrated our sensor data with Influx Database (InfluxDB), being a time-series optimized database toolkit that contains an all-in-one package of dashboards, queries, tasks, and agents. InfluxDB includes a User Interface (UI) that features a Data Explorer, dashboarding tools, and a script editor, enabling a multi-tenanted time-series, background processing, and monitoring agent, thus, providing a flexible deployment and setup suitable for storing IAQ measurements. As depicted in Figure 12, we can use the integrated Data Explorer to quickly browse through the metric and event data collected from the sensor nodes from our experimental LoRaWAN network and apply common transformations.

### 3.5. Front-End Application Server

**Figure 13:**
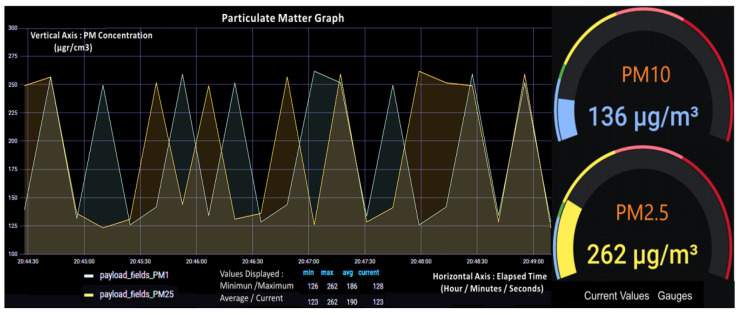
Visualization of Indoor Air Quality data by Grafana server using the Sensirion SPS30 sensor readings. (**Left**) Time-based graphic recording of PM values for the depicted time interval of 5 min, including minimum, maximum, average, and current values. (**Right**) current gauge values of PM_2.5_ and PM_10_ in μg/cm^3^ to be divided by a factor of 10.

Finally, data that has been stored into InfluxDB is integrated with a Grafana server making use of a front-end application that permits data visualization through this server. We use this server because it includes a built-in Graphite query parser, which allows us to query, visualize, alert on and understand our metrics irrespective of where they are stored. Figure 13 depicts PM_2.5_ and PM_10_ Indoor Air Quality data visualization provided by the Grafana server.

## 4. Conclusions

The goal of the present study being the development of a firm, highly secured, prominently low-powered, open-source, low cost and scalable wireless LoRaWAN network, for IAQ monitoring in real-time was achieved. Such an applicable smart wireless sensory network with an IoT platform infrastructure, for indoor air quality management purposes, could be promising to minimize airborne infectious disease, such as COVID-19, transmission and increase comfort and productivity. The platform implementation overall, emphasized low-cost and open-source architectures. Realizing such a smart, expandable, and cost-effective wireless network of air quality sensors could contribute to de-escalating levels of indoor particulate air pollution. Thus, decreasing COVID-19′s contagion, and risk of COVID-19 infections while leading to fewer lung infections, decreased respiratory symptoms, and less chronic obstructive pulmonary disease, cardiovascular diseases, or even lung cancer as posed by the World Health Organization.

Such a feasible, independent, and expandable long-range wireless sensor network that includes low consumption, long-lasting completely wireless sensors, and a smart platform with analytics for air quality management, would enable users to manage all air quality-related risks inside buildings and offices. The simulation we implemented proved to be able to monitor air quality in buildings with great success leading to improved breathing air within premises.

Regarding infrastructure capacity, the proposed system could also send signals to existing HVAC equipment to control ventilation by itself to avoid the risk of spreading airborne infections. Over time, cumulative data can be used to add to analytics in the building related to air quality. LoRaWAN schemes use end-to-end AES128 bit security encryption to ensure and enhance data security transmission on the whole.

The above-described wireless sensor network implementation proved to be reliable and robust according to the simulation results. The fewest number of lost packets occur by installing the gateway at the geometrical center of the building. This can be explained by simple observation of the topology of the nodes-gateway in the simulation, as the packets in this scenario usually have to penetrate fewer walls to reach the gateway than in the other two scenarios. No consideration of any kind was given to metallic, wooden, or plastic equipment being present in the premises, which would otherwise contribute even further to propagation losses. Given that this assumption holds for all three of the mentioned scenarios, we can assume that the installation of the gateway in the geometric center of a building will result in the fewest packet losses for the network in three cases. This should provide a reliable LoRa Wireless Area IAQ Sensor Network on the whole.

According to the specifications provided by the manufacturers, the vast majority of sensors tested provide reliable and accurate measurements for gases, PM, and other natural values, while some even combine these measurements to calculate the IAQ factor directly. Exposure to chemicals, aging of hardware, and deviation of measurement accuracy are taken into account, concerning the exposure to adverse environmental conditions. Their small size combined with their low weight and low cost are constructively combined for more advanced, low-cost, autonomous, and lightweight air quality control devices as required in IoT applications. All IAQ sensor nodes are to be placed indoors where a continuous power supply is vastly available while connected to the local energy network. So, energy consumption is not an issue, sensor nodes may not rely on batteries, although the sensors and LoPy platforms target low-power appliances according to specifications while utilizing low consumption strategies. Such sensors can be used in inexpensive implementations. Independent low-cost platforms with specific peripherals or devices and software applications may be combined in IoT sensor implementations as they adopt standard connectivity technologies, such as I2C, SPI, or UART. Concluding, as it regards the tested sensors, we can say that there are commercially available off-the-shelf (COTS) sensors that meet the needs for accuracy and deviation of measurements; resistance to the influence of environmental factors; price; a variety of measured quantities of pollutants; small size and weight; standard connectivity; and independence from specialized hardware and software for their operation.

Therefore, it is possible to construct a variety of Indoor Air Quality monitors for keeping track of PM_2.5_, PM_10_, and TVOC’s to meet World Health Organization guidelines by 2030.

Regarding future work, we will be working on system development and improvement, by adding other air quality sensors, to collect and monitor as much data as possible in future work. This will allow us to do data analysis and end up with accurate conclusions about the IAQ of larger buildings, like schools and hospitals. At the same time, simulation tools, such as Network Simulation 3 (NS3), will help us compare the theoretical results, with those that arise from our real-life experiments. We aim to isolate the elements that affect infections, such as COVID-19, and built an EWS based on low-cost sensors, to warn people about IAQ and airborne infections and prevent further contagion.

## Figures and Tables

**Table 1:** Sensors for IAQ measurements.

Sensors	Manufacturer	Datasheet
Sensirion SVM30	Sensirion	https://www.sensirion.com/en/environmental-sensors/multi-gas-humidity-and-temperature-module-svm30/ (accessed on 12 July 2021).
Renesas ZMOD4410	Renesas	https://www.renesas.com/us/en/products/sensor-products/gas-sensors/zmod4410-indoor-air-quality-sensor-platform (accessed on 15 July 2021).
BOSCH BME680	Bosch	https://www.bosch-sensortec.com/products/environmental-sensors/gas-sensors/bme680 (accessed on 5 August 2021).
Sensirion SPS30	Sensirion	https://www.sensirion.com/en/environmental-sensors/particulate-matter-sensors-pm25/ (accessed on 3 August 2021).
B5W-LD0101-1/2	Omron	http://components.omron.eu/Product-details/B5W-LD0101-1_2 (accessed on 10 August 2021).

**Table 2:** Sensor characteristics and performance. Unit ppm is an abbreviation for “parts per million” describing dilute solutions in chemistry also expressed as milligrams per liter (mg/L). Accordingly, ppb stands for “parts per billion” that corresponds to 1 μg/L.

Sensor	Type	Measured Parameter	Range	Resolution
Sensirion SVM30	multiple metal-oxide sensing elements—the pixels—on one chip	Gas sensing	0–1000 ppm For the indoor specified range: 0.3–30 ppm 0.5–3 ppm	0.2% of a measured value
		Ethanol		
		H_2_ signal		
		H_2_		
		Ethanol Signal		
		Air Quality TVOC signal	0–60,000 ppb	0–2008 ppb 1 ppb
				2008–11,110 ppb 6 ppb
				11,110–60,000 ppb 32 ppb
		Air Quality TVOC signal	0–60,000 ppb	0–2008 ppb 1 ppb
				2008–11,110 ppb 6 ppb
				11,110–60,000 ppb 32 ppb
		Air Quality CO2eq signal	400–60,000 ppm	400–1479 ppm 1 ppm
				1479–5144 ppm 3 ppm
				5144–17,597 ppm 9 ppm
				17,597–60,000 ppm 31 ppm
		Hum. Meas. Range	0–100% RH	Recommended in range 25–75%
		Temp. Meas. Range	−20–85 °C	Recommended in range 5–55 °C
Renesas ZMOD4410	Resistor heat—Low-rate Minimum 3 s	TVOC	0–1,000,000 ppb	For IAQ
			For IAQ 160–1000 ppm	160–1000 ppm
		eCO2	400–5000 ppm	
		Smell/odors	Sulfur based or organic based-acceptable	
BOSCH BME680	direct output of IAQ Index for Air Quality index	Pressure	300–1100 hPa	
		Humidity Temperature	0–100%	
			40–85 °C	
Sensirion SPS30	Optical Laser light Source	Mass concentration	1–1000 μg/m^3^	1 μg/m^3^
			PM_1.0_, PM_2.5_, PM_4_, PM_10_	0.3–1.0 μm, 2.5 μm, 4.0 μm, 10.0 μm
		Particle detection size Number# concentration	0–3000 #/cm^3^	0.3–0.5 μm, 0.3–1.0 μm, 0.3–2.5 μm, 0.3–4.0 μm, 0.3–10.0 μm accordingly
			PM_0.5_, PM_1.0_, PM_2.5_, PM_4_ and PM_10_	
Omron B5W-LD0101-1/2	LED light source Light Scattering sensing method	Particle detection size range	Vout1 = 0.5–2.5 μm	
			Vout2 ≥ 2.5 μm	

**Table 3:** Sensors’ accuracy and precision.

Sensor	Accuracy Consistency	Precision	Drift
Sensirion SVM30	typical: 1.3% of the measured value	<0.25% RH/year	<0.02 °C/Year
Renesas ZMOD4410	±25%	±25% no external calibration	Durability to Siloxanes Change in sensitivity ±5%
		±15% with external calibration	
BOSCH BME680	Response time (τ 33–63%)	<1 s (for new sensors)	±4%
	Sensor-to-sensor deviation	+/− 15% +/− 15	
	RMS Noise Sensitivity Error Temp coefficient offset	0.12 Pa (Equivalent to 1.7 cm)	
		±0.25% (Equivalent to 1 m at 400 m height change)	
		±1.3 Pa/K (Equivalent to ±10.9 cm at 1 °C temp. change)	
	±3% relative humidity	≤1.5% relative humidity hysteresis	
Sensirion SPS30		Precision PM_1_, PM_2.5_ = ±10 μg/m^3^ @ 0–1000 μg/m^3^	0–100 μg/m^3^ = ±1.25 μg/m^3^/year
		PM_4_, PM_10_ = 0–100 μg/m^3^ = ±25 μg/m^3^ 100–1000 μg/m^3^ ± 25% m.v	100–1000 μg/m^3^ = ± 1.25% m.v./year
		0–1000 #/cm3 = ±100 #/cm3	0–1000 #/cm^3^ ± 12.5 #/cm^3^/year
		1000–3000 #/cm3 = ±10% m.v	1000–3000 #/cm^3^ ± 1.25% m.v./year

**Table 4:** Sensors’ communication and energy characteristics.

Sensor	Communication Interface	Voltage	Power Consumption	Sampling Rate
Sensirion SVM30	I^2^C	5 V	245 mw/49 mA	80 ms 14 Hz
				400 Hz max
				1 s 1 Hz
Renesas ZMOD4410	I^2^C	1.8–3.6 V	23 mW/16 mA max	Minimum 3 s
			I sleep = 450 nA	10 s typical
BOSCH BME680	ISP UART	5 V	Sleep mode 3.7 µA at 1 Hz humidity, pressure, and temperature 0.09–12 mA for p/h/T/gas depending on the operation mode	8 s
Sensirion SPS30	I^2^C only Mass/UART (full spec measurement)	4.5–5.5 V	275 mW/55 mA or	1 s 1 Hz sample
			1.65 mW/330 μA idle	
			0.19 mW/38 μA sleep	
Omron B5W-LD0101-1/2	Pulsed output	5 V	450 mW/90 mA	10-s min 20 s optimum
		Vripple <30 mV		

**Table 5:** IAQ sensors’ price information.

Sensors	Cost
Sensirion SVM30	19.49 €
Renesas ZMOD4410	6.95 €
BOSCH BME680	22.00 €
Sensirion SPS30	38.83 €
Omron B5W-LD0101-1/2	12.87 €
